# Tracheostomy for the pediatric patient with fibrodysplasia ossificans progressiva: a case report

**DOI:** 10.1186/s40792-024-01864-3

**Published:** 2024-03-15

**Authors:** Megumi Kobayashi, Misako Hirai, Makoto Suzuki, Akira Sasaki

**Affiliations:** 1 Department of Surgery, School of Medicine, Iwate Medical University, 2-1-1 Idaidouri, Yahaba, Shiwa, Iwate, 028-3695 Japan; 2 Ibaraki Welfare and Medical Center, 1872-1 Motoyoshida, Mito, Ibaraki 310-0836 Japan

**Keywords:** Fibrodysplasia ossificans progressiva, Tracheostomy, Surgical intervention, Quality of life, Flare-ups, Dyspnea

## Abstract

**Background:**

Fibrodysplasia ossificans progressiva (FOP) is an extremely rare connective tissue disease characterized by subsequent ossification of skeletal muscles, tendons, ligaments, and other fibrous tissues. The ossification of these tissues progresses during childhood and leads to limb and trunk deformities. Since any surgery may trigger subsequent ossification, it is relatively contraindicated for patients with FOP. In this report, we describe our experience in performing tracheostomy in a pediatric patient with FOP who developed a restrictive respiratory disorder due to progressive deformity of the trunk.

**Case presentation:**

A 12-year-old boy, diagnosed with FOP at the age of one, was referred for a tracheotomy after requiring 2 months of oral intubation and mechanical ventilation due to severe deformity-induced dyspnea. After changing from oral intubation to nasal intubation, we carefully considered the indications and benefits of tracheostomy in patients with FOP. Eventually, tracheostomy was successfully performed using our surgical design: creating a skin incision at the level of the cricoid cartilage that can always be identified, creating inverted U-shaped incision on the anterior tracheal wall to make a flap, and suturing the entire circumference of the tracheotomy and skin. One month after the surgery, he regained normal breathing and pronunciation and returned to school. The patient showed no unfavorable postoperative outcomes over a 4-year follow-up period.

**Conclusions:**

Tracheostomy in our pediatric case of FOP required careful perioperative management. However, it could effectively improve the patient’s quality of life.

## Background

Fibrodysplasia ossificans progressiva (FOP) is an extremely rare connective tissue disease associated with mutations in the *activin A receptor type 1* gene. FOP affects approximately 1 in 2 million people, with only 60–80 cases identified in Japan [1]. It is characterized by tissue swelling (flare-ups) and subsequent ossification of the fascia, muscles, tendons, ligaments, and other fibrous tissues beginning from childhood, consequently leading to deformity of the limbs and trunk. The first “flare-up,” which leads to formation of bones associated with FOP, usually occurs before the age of 10. The period from FOP diagnosis to the occurrence of early symptoms is approximately 1.5 years [1]. Regarding flare-ups occurrence, steroids and bisphosphonates are generally used for preventing ossification. However, the efficacy of these drugs remains uncertain.

FOP does not affect intelligence. The average life expectancy of patients with FOP is 40 years. The main causes of death are pneumonia and cardiopulmonary failure due to thoracic insufficiency syndrome [2]. Any form of physical trauma to the body such as a knock, bump, fall, intramuscular vaccination, surgery, or even a common viral infection such as influenza can trigger activation of the FOP gene.

In this report, we describe our experience in performing tracheostomy in a pediatric patient with FOP who developed restrictive respiratory disorder due to progressive deformity of the trunk from early childhood.

## Case presentation

A 12-year-old boy (height: 109 cm; weight: 21 kg) with FOP was referred to our hospital for tracheostomy. He had undergone orotracheal intubation 2 months prior owing to restrictive ventilatory impairment caused by severe deformities and contractures of the neck, thorax, and spine.

The patient was diagnosed with FOP at the age of one. The ectopic ossification and deformation of his cervical scapulothoracic cage occurred and progressed without intellectual disability, difficulties in walking, or problems attending school. He required oxygen administration from the age of nine years because of restrictive ventilation failure. However, ectopic ossification and his cervical scapulothoracic deformity rapidly progressed. At the age of 12, he developed severe dyspnea requiring emergency oral intubation and mechanical ventilation.

**Fig. 1:**
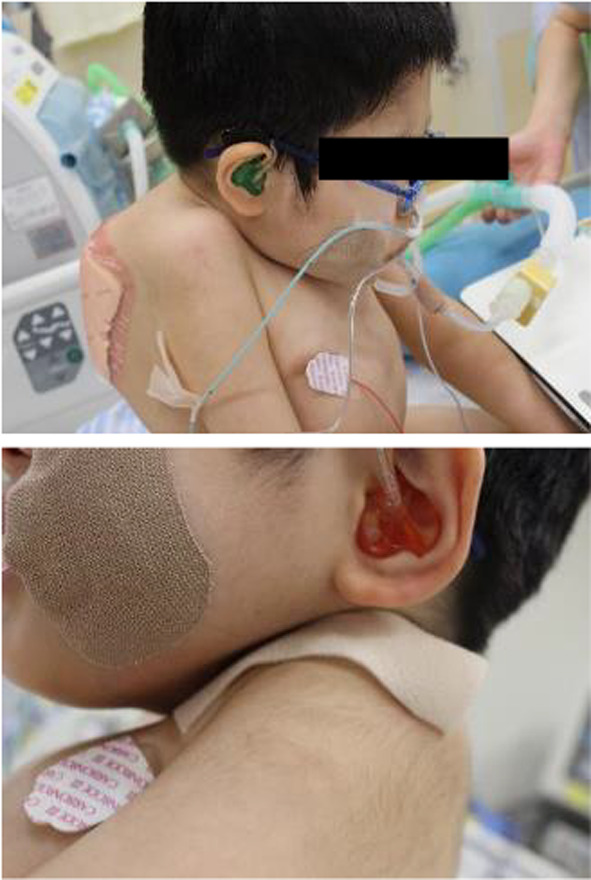
Limited space in the anterior neck due to thoracic trunk deformity at initial oral intubation

We were concerned that many surgical procedures, including tracheostomy, were typically contraindicated for patients with FOP, because they could lead to ectopic ossifications. Therefore, we visited the patient at the referring hospital for preliminary assessment. Physical examination revealed that the patient could not be placed in the supine position. In addition, we observed that the space in his anterior neck was insufficient for the surgery because the patient could not stretch his neck (Fig. 1). Based on these findings, tracheostomy was considered extremely difficult for him.

Very few cases of tracheostomy performed in patients with FOP during childhood have been reported. While we were researching surgical options for our patient, unscheduled extubation was performed twice within 4 weeks. Each reintubation after the events was very difficult. Furthermore, the patient had difficult experiences such as flare-ups, atelectasis, and pneumonia. He endured great emotional distress and hoped for tracheostomy as soon as possible. After thorough research and several preoperative meetings, we decided to perform the procedure.

**Fig. 2:**
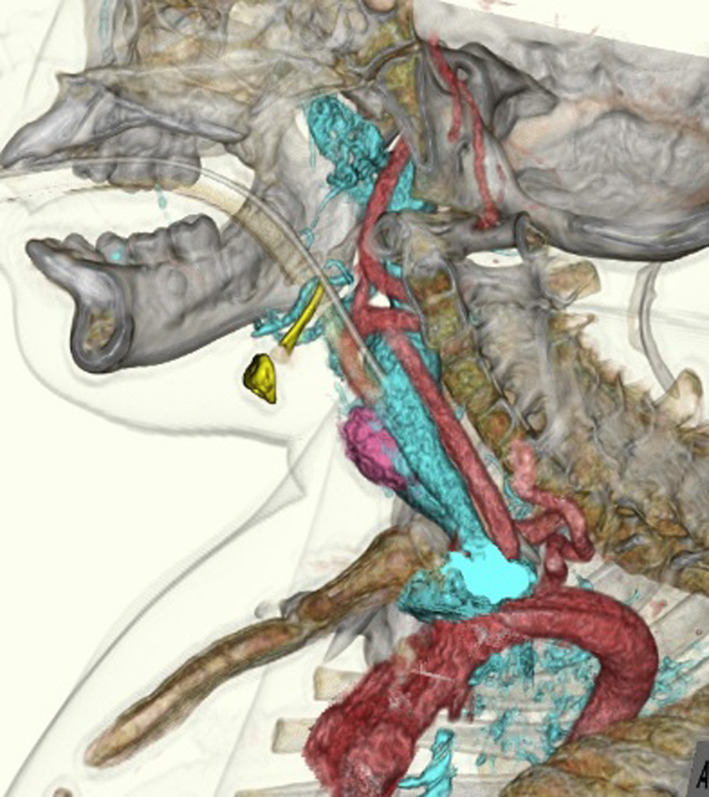
Three-dimensional computed tomography (3D-CT) before tracheostomy

Two weeks before surgery, the patient was transferred to our hospital for preoperative evaluation and preparation. We examined the neck and thorax deformity under general anesthesia and changed the intubation route from oral to nasal. Three-dimensional computed tomography (3D-CT) was performed to facilitate the selection of his tracheostomy tube (Fig. 2). Given the patient’s severe scoliosis and contracture, the shape and condition of his neck and chest and the position of his trachea were significantly different from those of a healthy child. Therefore, preoperative 3D-CT was used to clearly visualize the space from the anterior chest wall to the neck, measure the distance from the neck skin to the anterior tracheal wall, and determine the meandering course of the trachea in his daily posture. These insights provide valuable in preparing a tracheal tube tailored to the patient’s specific needs, accounting for the appropriate thickness, length, and shape in advance. Ensuring the insertion of a suitable tube during tracheostomy was also important for the patient’s comfort in the early postoperative period.

**Fig. 3:**
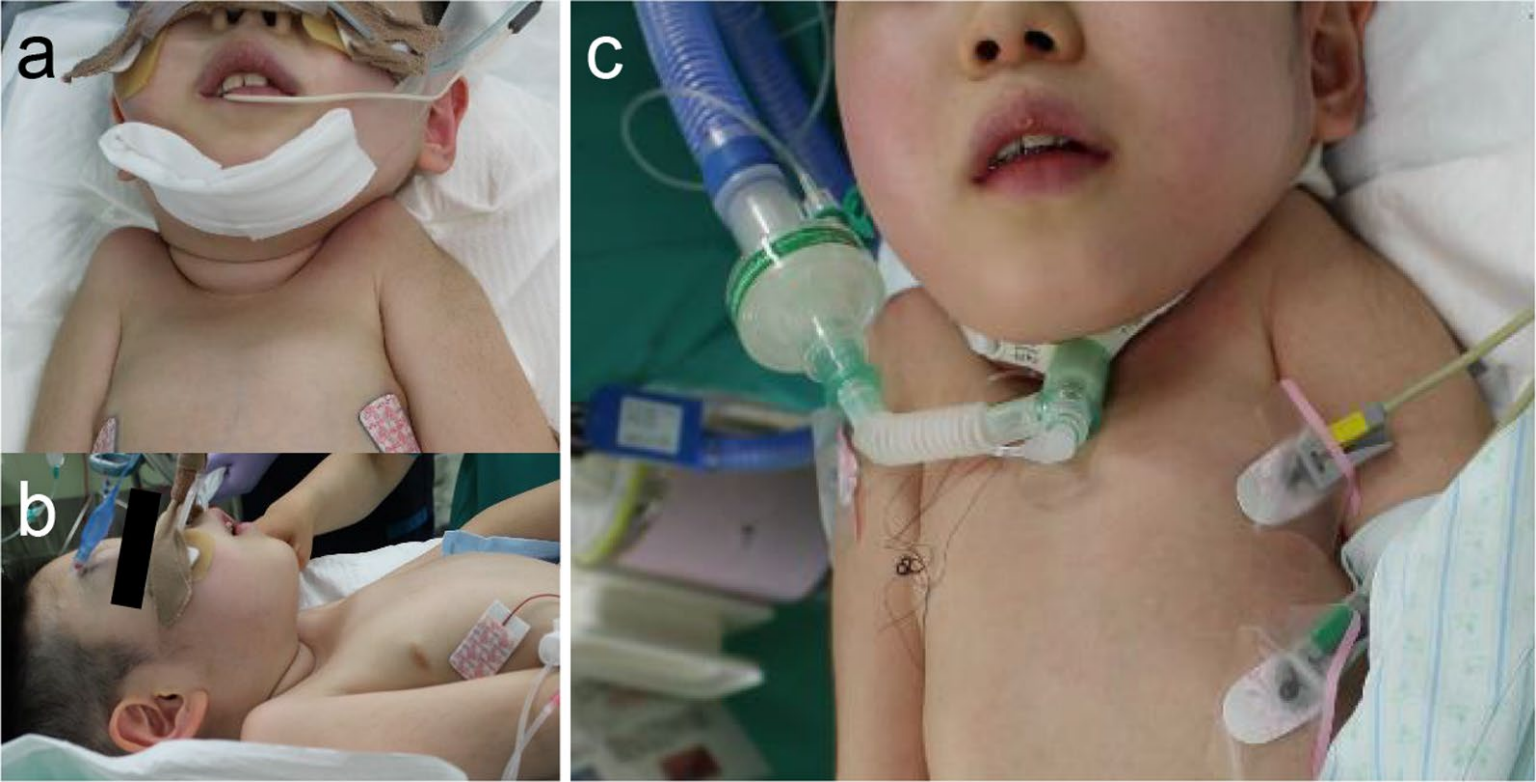
Anterior neck appearance during tracheostomy under general anesthesia. **a**, **b** Anterior neck space is available in maximum neck extension position; **c** A 5.5 Fr cuffed tracheostomy tube is inserted after tracheostomy

**Fig. 4:**
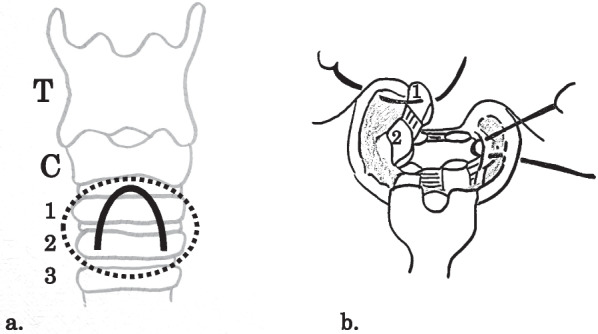
Schematic diagram showing an inverted U-shaped tracheal wall incision and the anterior tracheal wall flap. **a** Inverted U-shaped tracheal wall incision. The dashed circle indicates the surgical field of view. T: thyroid cartilage, C: cricoid cartilage, 1–3: tracheal cartilage; **b** Anterior tracheal wall flap, 1.2: tracheal cartilage (lateral view)

Although the patient was administered high-dose steroids for preventing flare-ups, we performed the tracheostomy procedure cautiously. Unlike typical tracheostomy, the trachea was far from the skin because the patient’s neck could not be tilted. A horizontal incision was made on the skin at the level of the inferior border of the cricoid cartilage, then the anterior cervical musculature was divided along the midline gently. The blood vessels in the thyroid isthmus were separated from the tracheal surface as far as possible without damaging thyroid tissues. An inverted U-shaped incision was made in the tracheal wall from the lower part of the cricoid cartilage to the upper part of the second tracheal cartilage. Thereafter, the skin was sutured to the entire circumference of the tracheal hole. This surgical method, involving suturing the trachea to the skin, especially closing the mediastinal side with a tracheal wall flap, allowed for safe reinsertion immediately after surgery, even if the tube was removed unexpectedly. To prevent additional chronic trauma, we did not suture the tube to the skin. In addition, his neck and shoulder does not move freely, so even if the cannula is not tightly secured to his neck, unplanned removal is unlikely unless the cannula is intentionally pulled out. We performed bronchoscopy to confirm a suitable tube for the patient’ deformed trachea and finally selected a 5.5 Fr cuffed tracheostomy tube (Figs. 3 and 4).

The patient developed postoperative posterior reversible encephalopathy syndrome (PRES), which appeared to be caused by stress associated with long-term intubation, requiring sedation and mechanical ventilation for 2 days. He had high blood pressure due to PRES for a week, without experiencing other complications. Oral intake was initiated on postoperative day 5, then he recovered to a good state in terms of activities of daily living. On postoperative day 7, the patient was transferred to the previous hospital and discharged after a period of physical therapy.

**Fig. 5:**
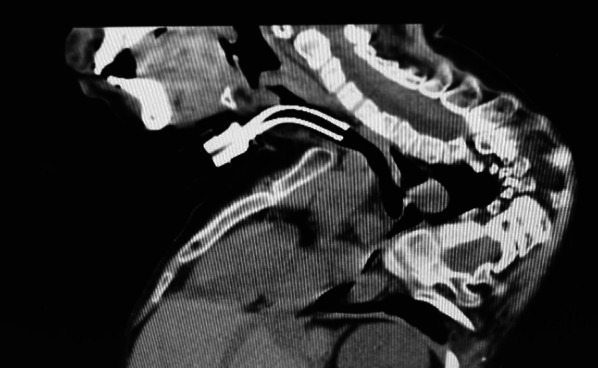
Computed tomography (CT) image at 1 month after tracheostomy. CT shows no ossification at the surgical site or abnormalities in cannula placement

One month after the surgery, the patient was placed on a ventilator at night only. The tracheostomy cannula was replaced with the cuffless one, consequently, he could breathe and speak easily. The postoperative CT showed no ossification at the surgical site or abnormal cannula placement (Fig. 5). The patient gradually regained the ability to speak and could return to his school. His ability to resume his daily activities indicated that the tracheostomy improved his quality of life (QOL).

At the time of this report, the patient was 16 years old. He is a high school student and has lived with his family as he wanted before the surgery. Since the patient is growing with a progressive disease, several problems have been encountered. His ventilatory impairment progressed to the point where he needed a ventilator almost all day long, and required high airway pressure with a cuffed cannula to maintain breathing during sleep. Currently, it is difficult for him to undergo cannula replacement without general anesthesia due to pain and fear of dyspnea.

## Discussion

There are very few reports of tracheostomy performed in patients with FOP; thus, the prognosis of the procedure remains unclear. Respiratory failure may occur in pediatric patients with FOP with rapid progression of heterotopic ossification and deformation. We believe that careful management with tracheostomy would be useful for maintaining patients’ QOL.

According to the treatment guidelines provided by the International Fibrodysplasia Ossificans Progressiva Association for preserving the respiratory tract in emergency situations, nasotracheal intubation should be the preferred and initial treatment option. However, tracheostomy may be performed if tracheal intubation was difficult [3]. This approach is supported by several reports on the possibility of FOP flare-ups occurring after minor stimulation [4–7]. In our case, we describe a case of a smart 12-year-old boy enjoying school life. Therefore, his family and medical team staff could not give up on his engagement in normal daily activities while keeping the tracheal intubation.

A few reports on tracheotomy in patients with FOP indicated that muscle injury could cause ossification, whereas damage to the skin or subcutaneous tissue did not trigger it [8–10]. Other case reports have shown that multiple laparotomies did not cause ossification [11]. Additionally, the author of a previous case report advised that we opt for safe tracheostomy performed after preoperative 3D-CT [12]. These reports supported our decision in choosing the option of tracheostomy. Furthermore, our treatment was based on our previous experience with performing tracheostomies in patients with severe motor and intellectual disabilities, who had neck contracture and meandering trachea caused by severe scoliosis and chest deformity. This experience also helped us consider postoperative cannula management.

Similar to the patients with FOP who require endotracheal intubation [13, 14], our patient in the present study had a severe physical deformity. Consultations with healthcare staff from multiple disciplines confirmed using high-dose steroids for preventing flare-ups in the perioperative period. Owing to the patient’s severe deformity and limited backward flexion of his neck, we could not perform the typical operative procedures and techniques. Eventually, the tracheostomy was successfully performed under our surgical design. The procedure was also effective in preventing postoperative dislocation of the tracheal cannula and facilitating reinsertion after extubation, at any condition. We were also careful to ensure that invasion into the fascia and muscles during surgery was as minimal as possible, even when using retractors. After the surgery, no ossification occurred at the surgical site for four years. More importantly, the patient has been satisfied with his postoperative QOL. The outcome of this case indicated that our experience with tracheostomy could provide useful information for managing pediatric patients with FOP with early-onset dyspnea.

Nevertheless, due to the patient’s severe physical deformity, several problems occurred during his postoperative management and care. Existing pediatric tracheostomy cannulas were not suitable for this case who required long vertical insertion through the tracheostomy due to tracheal deviation associated with severe scoliosis. The tip of the tracheostomy tube had to be placed vertically in his tracheobronchial space. Repeatedly performing bronchoscopy and re-selecting the cannula depending on the condition in the trachea and his growth were important.

For his family, daily care has been challenging in terms of fixing the cannula band, cleaning the neck skin, avoiding skin ulceration by the cannula and accessories, and preventing unplanned removals. However, his family was grateful and very happy to be able to have him live at home with them.

Since the patient is now an adolescent, his body is growing rapidly, the scoliosis is progressing, and his trachea is deviating in a complicated manner. Because his lung disease (thoracic insufficiency syndrome) has progressed gradually and required constant positive pressure ventilation, he currently uses a cuffed tube. Therefore, cannula selection has become quite challenging. We will continue to report the long-term outcomes of this case while considering the optimal cannula at each follow-up stage.

## Conclusions

Tracheostomy could be a useful option for improving the QOL in a patient with FOP during childhood.

## Data Availability

All data generated or analyzed during this study are included in this published article.

## Abbreviations

FOP: Fibrodysplasia ossificans progressive
3D-CT: Three-dimensional computed tomography
PRES: Postoperative posterior reversible encephalopathy syndrome
QOL: Quality of life
